# Psychometric validation of the revised SCOPA-Diary Card: expanding the measurement of non-motor symptoms in parkinson's disease

**DOI:** 10.1186/1477-7525-9-69

**Published:** 2011-08-18

**Authors:** Regina Rendas-Baum, Philip O Buck, Michelle K White, Jane Castelli-Haley

**Affiliations:** 1 QualityMetric Inc., Lincoln, RI, USA; 2 Teva Neuroscience, Inc., Kansas City, MO, USA

**Keywords:** Parkinson's disease, quality of life, SCOPA, diary, reliability, validity, non-motor symptoms

## Abstract

**Background:**

To identify key non-motor symptoms of Parkinson's disease (PD) to include in a daily diary assessment for off-time, revise the Scales for Outcomes of Parkinson's disease Diary Card (SCOPA-DC) to include these non-motor symptoms, and investigate the validity, reliability and predictive utility of the Revised SCOPA-DC in a U.S. population.

**Methods:**

A convenience sample was used to recruit four focus groups of PD patients. Based on findings from focus groups, the SCOPA-DC was revised and administered to a sample of 101 PD patients. Confirmatory factor analysis was conducted to test the domain structure of the Revised SCOPA-DC. The reliability, convergent and discriminant validity, and ability to predict off-time of the Revised SCOPA-DC were then assessed.

**Results:**

Based on input from PD patients, the Revised SCOPA-DC included several format changes and the addition of non-motor symptoms. The Revised SCOPA-DC was best represented by a three-factor structure: Mobility, Physical Functioning and Psychological Functioning. Correlations between the Revised SCOPA-DC and other Health-Related Quality of Life scores were supportive of convergent validity. Known-groups validity analyses indicated that scores on the Revised SCOPA-DC were lower among patients who reported experiencing off-time when compared to those without off-time. The three subscales had satisfactory predictive utility, correctly predicting off-time slightly over two-thirds of the time.

**Conclusions:**

These findings provide evidence of content validity of the Revised SCOPA-DC and suggest that a three-factor structure is an appropriate model that provides reliable and valid scores to assess symptom severity among PD patients with symptom fluctuations in the U.S.

## Background

Parkinson's disease (PD) is the second most prevalent neurodegenerative disease in the U.S., afflicting about one million Americans over age 60 [1]. Motor symptoms associated with PD include bradykinesia (slowness of movement), tremor of resting muscles, postural instability or impaired balance, and gait disturbances [2]. In addition to motor symptoms, a wide range of non-motor symptoms are also associated with PD. The most common include neuropsychiatric symptoms (depression, anxiety, cognitive impairment, etc.), sleep dysfunction, autonomic dysfunction (bladder dysfunction, excessive sweating, etc.), gastrointestinal dysfunction (constipation, hypersalivation, difficulty swallowing, etc.), and sensory symptoms (pain, olfactory dysfunction) [3-9].

PD treatment typically targets dopamine replacement with levodopa and agents to improve its bioavailability [10]. However, after several years of dopaminergic therapy, most patients experience fluctuations between "off-time" and "on-time" in both motor and non-motor symptoms as much as 2-3 hours a day [11]. Off-time refers to periods when PD symptoms return despite medication. Conversely, on-time refers to periods when PD medications are working well and symptoms are under control [12-14]. Off-time may be predictable, such as the recurrence of symptoms preceding a scheduled medication dose (often referred to as "wearing off") [14,15]. Sometimes, however, spontaneous symptoms may recur unrelated to nearing next medication dose [14].

Assessing off-time is important for researchers as well as clinicians, who monitor off-time for needed changes in medication schedule, dose, or additional treatments needed [14,16]. Off-time events vary in duration, intensity, frequency, and timing. As such, measuring off-time requires a daily diary format, which may provide a more accurate reflection of changing clinical status for fluctuating symptoms than static instruments [12,17]. Diary format assessments are most practical when designed to be patient self-administered. There are many widely used assessments available to measure motor symptom severity associated with PD, and to a lesser extent non-motor symptom severity [4,12,18], but these tools are predominantly static, lacking the ability to measure symptom fluctuations [11]. One recent review [12] found no currently available patient-reported daily diary that would measure both fluctuating motor and non-motor PD symptoms, despite a breadth of literature citing the tremendous debilitating impact of fluctuating symptoms [11,19-21].

Currently, the Scales for Outcomes of Parkinson's disease Diary Card (SCOPA-DC), a seven time-point assessment of motor symptoms, is the only validated daily diary instrument designed to measure both motor symptom severity and motor symptom fluctuations in PD patients [22]. The SCOPA-DC was validated in a sample of cognitively unimpaired PD patients recruited from Leiden University Medical Center, in the Netherlands, and has not been validated in the U.S. In addition, non-motor symptoms are absent from the SCOPA-DC, thus missing an increasingly important area of PD symptomatology [7]. The intent of this study was to identify key non-motor symptoms to include in a daily diary assessment for off-time, revise the SCOPA-DC to include these non-motor symptoms, and investigate the validity, reliability and predictive utility of the Revised SCOPA-DC in a U.S. population.

## Methods

### Phase I - qualitative study

Phase I of the study consisted of several steps designed to: 1) establish the content validity and appropriateness of the (original) SCOPA-DC in a U.S. population; 2) determine the feasibility of adding new items/domains that measure non-motor functions; 3) evaluate the content validity of the Revised SCOPA-DC in a U.S. population.

#### Literature review

The first step in the process of refining the SCOPA-DC was to gain a sound understanding of the full spectrum of PD symptoms, their interconnections and impact on Health-Related Quality of Life (HRQOL), and association with off -time. The literature review phase further served to help define inclusion and exclusion criteria for the study, and to develop interviewer guide materials for the focus groups. PubMed was searched using text words: "rating scale", "non-motor", "nonmotor", "daily diary", "on/off", "on-off", "quality of life", or "SCOPA" in combination with ''Parkinson's", or ''Parkinson". Abstracts were reviewed for content and reference lists were used to obtain additional relevant references.

#### Focus groups

Focus groups took place between April and May, 2009 across 3 locations in the U.S. A convenience sample was used to recruit PD patients for 3 initial focus groups (N = 8 per focus group) and one cognitive debrief focus group (N = 9) according to the following criteria: age 30 and older; off-time symptoms between 1% and 25% of the day or more; at least 2 of the following 3 PD symptoms on a typical day: 1) slowed ability to start and continue movements, 2) resting tremors or shakiness, and 3) rigidity or inability to complete a movement; currently on dopaminergic therapy; never had brain surgery to treat PD; fluent in English.

The first 3 focus groups were used to assess the original SCOPA-DC for comprehension and validity, obtain general information on patients' experience with PD symptoms, including their ability to identify when they were experiencing off-time, and to elicit non-motor symptoms considered to be important to patients with off-time. The first part of the 90-minute focus groups followed a discussion guide but elicited open discussion. Mid-way through the groups, patients were asked in more detail about the non-motor symptoms that occur with off-time, including giving examples of off-time experiences and how these impacted their lives. Patients were then asked to rate the importance of all the symptoms listed that occurred with off-time. Finally, patients were asked about meaning, relevance, and clarity of the original SCOPA-DC, including the instructions, item content, and response options.

The last focus group was a cognitive debrief [23] of the Revised SCOPA-DC. The interviews followed a discussion guide developed specifically for the SCOPA-DC evaluation and cognitive review. Ethics approval was granted by the New England Institutional Review Board (NEIRB) and written consent was obtained prior to interviews.

### Phase II - psychometric evaluation of the Revised SCOPA-DC

#### Study design

##### Recruitment

The psychometric evaluation of the Revised SCOPA-DC was a cross-sectional, non-randomized study that surveyed non-institutionalized adults age 30 and older with self-reported doctor-confirmed PD. Patients were recruited online through Knowledge Networks' (KN) Health Profile panel [24], between October and December, 2009. The recruitment and baseline data collection was followed by an at-home data collection effort which included the completion of the Revised SCOPA-DC over the course of 3 consecutive days. The following inclusion criteria were applied: 1) ever experienced resting tremors and at least one of the following symptoms due to PD: slowed ability to start and continue movements; rigidity or inability to complete a movement; difficulty with balance or instability; stooped, forward-leaning posture; freezing or sudden, brief inability to move the feet; 2) willing to provide informed consent. A subject was excluded if either of the following applied: 1) self-reported history of brain surgery to treat PD; 2) declined consent. PD patients who were eligible to participate in the study were mailed study packets containing: study instructions; 2 copies of the informed consent; instructions on how to identify off-time; an instructional DVD on how to complete the Revised SCOPA-DC; 5 copies of the Revised SCOPA-DC; an end of study questionnaire to capture patients' feedback on participating in the study; a prepaid return envelope to mail completed forms. Ethics approval was granted by the NEIRB.

##### Study measures

General demographic information was collected online. Specific information on clinical characteristics included questions regarding the type of PD symptoms they experienced, time since PD diagnosis, types of PD treatments and whether they experienced off-time. In addition, the following instruments were used in the online portion of the study: 1) Short Form-12 version 2 (SF-12v2) [25], a general HRQOL instrument that consists of 12 items from which two composite measures can be derived: the Physical Component Summary (PCS) and the Mental Health Component Summary (MCS), measuring overall physical and mental health, respectively [25,26]; 2) Parkinson's Disease Questionnaire-8 (PDQ-8) [27], a questionnaire comprised of 8 questions about the physical and psychosocial impact of PD such as difficulty concentrating or dressing; 3) Wearing Off Questionnaire-9 (WOQ-9) [28], a 9-item survey that asks about the reduction of or improvement of motor and non-motor symptoms in relation to the timing of medication taking.

The following instruments were mailed to study participants to be completed in paper-and-pencil form: 1) Revised SCOPA-DC, a diary card to be completed 7 times per day for 3 consecutive days; 2) the end-of-study feedback questionnaire, a global debriefing of the participant's experience with completing the Revised SCOPA-DC that consisted of an open-ended question and 14 scaled and yes/no items.

##### Statistical analysis

#### Scoring of the Revised SCOPA-DC

Single-item scores were evaluated for the 11 symptom items in the Revised SCOPA-DC by summing responses over the 21 time periods. This 3-day sum score was transformed to a 0-100 scale, allowing for a maximum of 2 missing time periods per day.

Multi-item scores were evaluated for the three subscales derived from factor analyses by taking the average of the 3-day item scores for the items within the respective subscale. If at least one of the 3-day item scores was missing the subscale score was set to missing. In all cases, higher scores indicate greater difficulty, while the complement reflects "good functioning."

The coefficient of variation (CV) and standard deviation (SD) were used as measures of the stability of symptoms experienced by patients. The CV was evaluated by dividing the SD by the mean of the 21 time period scale scores. Similar to the original SCOPA-DC, [22] CV scores were not evaluated if the time period scale mean was below one. When the mean value is small, the ratio of these two quantities becomes unstable and its interpretation becomes difficult. While the CV is an informative statistic when the variability (stability of symptoms) tends to change with the mean (severity of symptoms), the sample SD is not affected by small mean values, which occurred frequently in our sample. Thus, in the current study both measures of variability were used to describe the stability of patients' symptoms.

#### Factorial structure of the Revised SCOPA-DC

It was hypothesized that the factorial structure of the Revised SCOPA-DC would be best represented by a 2-factor structure, corresponding to motor and non-motor symptom domains. One factor consisted of 4 items related to motor function and symptoms (walking, changing position, using your hands, uncontrollable movements) and the second factor consisted of the 7 non-motor items (feelings of exhaustion or fatigue, difficulty concentrating or remembering, feelings of anxiety or panic, unexplained pains, difficulty swallowing, frequent or urgent urination, sweating too much). Due to sample size (N = 101) limitations, the stability of results obtained from confirmatory and exploratory factor analyses was evaluated using a method akin to k-fold cross validation [29]. The sample was divided into 10 subsets of approximately equal size (9 subsets of size 10 and one of size 11) and factor analyses were carried out after exclusion of each of the ten subsets. Confirmatory factor analysis (CFA) was carried out to test the fit of the hypothesized 2-domain structure 10 times, with each subset removed in turn. CFA solutions were extracted using the robust maximum likelihood (MLR) estimator in Mplus 5.1 [30]. The CFA model fit was assessed using several indicators: comparative fit index (CFI), Tucker-Lewis Index (TLI), root mean square error of approximation (RMSEA) and standardized root mean residual (SRMR). Hu and Bentler's [31] guidelines were used to interpret the values of CFI and TLI (≥ .95), RMSEA (< .06) and SRMR (< .09) indicating close fit. If model refinement was deemed necessary, standard use of modification indices was undertaken [32], with a cutoff value of 10 [30].

Based on CFA results, exploratory factor analysis (EFA) was carried out with a maximum of 3 factors to explore alternative domain structures. EFA was conducted using the weighted least squares means and variance adjusted (WLSMV) under the specification of a censored normal distribution [30] for each of the items to account for distributional characteristics [32]. The promax rotation [33] was used to extract the number of factors. The recommended number of factors was based on goodness of fit indices (Chi-Square, RMSEA and SRMR) and the magnitude (≥ 0.4) of factors loadings. The final structure was recommended based upon the stability of factor loadings across the 10 runs. Upon determination of the number of factors that best represented the latent model of the Revised SCOPA-DC, CFA was carried out using the entire sample of 101 observations.

#### Item-level psychometrics

Item-total correlations (corrected for overlap) were evaluated by calculating the Spearman correlation coefficient between the subscale total and the 3-day item score. Item-total correlations ≥ 0.40 and small (< 10% increase) alpha-removed statistics were considered indicative of sufficient correlation with the underlying trait [34].

#### Reliability

Each subscale's coefficient alpha was interpreted against the standard criteria for sufficiency (≥ 0.80) [35]. Model-based reliability was also evaluated using unstandardized loadings and error variances obtained from the final CFA model [32].

#### Convergent and discriminant validity

Spearman correlations between the 3 Revised SCOPA-DC subscale scores and scores on the PDQ-8 Summary Index, the percentage of symptoms from the WOQ-9 and the SF-12v2 composite scores (PCS and MCS) were considered supportive of convergent validity if they were ≥ 0.40 [36]. Given the disease specific nature of the PDQ-8, it was hypothesized that Revised SCOPA-DC scores would be more strongly correlated with PDQ-8 and WOQ-9 scores than with SF-12v2 scores. Furthermore, the Psychological Functioning subscale would be more strongly correlated with MCS scores than with PCS scores and the Mobility and Physical Functioning subscales would be more strongly correlated with PCS scores than with MCS scores in order to be suggestive of discriminant validity.

#### Known-groups validity

Construct validity was examined using the framework of known-groups validity [37]. This type of analysis compares mean scale scores across groups known to differ on a clinical criterion measure. Groups were based on: 1) the presence or absence of off-time at baseline and 2) time since PD diagnosis (up to 5 years versus more than 5 years; 5 years was the sample median disease duration). Scale scores were compared across these groups and statistical significance was assessed using the independent samples t-test if the scores were normally distributed and the Mann-Whitney test otherwise.

#### Measurement of symptom fluctuations

It was hypothesized that patients who reported off-time at baseline would experience more symptom fluctuations than patients without off-time. Statistical significance of group differences in mean CV and SD scores was tested using the Mann-Whitney test. Significance testing was not conducted if the sample size was less than 5.

#### Prediction of off-time

Longitudinal binary logistic regression using generalized estimating equations (GEE) [38] was used to assess the relationship between single-period scale scores and the probability of experiencing off-time. The dependent variable was the binary response for off-time (for each time period), and the independent variable was the Revised SCOPA-DC score (for each time period). An exchangeable covariance structure was specified. The percentage of correctly predicted cases was evaluated using a cutoff probability ≥ 0.5.

## Results

### Phase I - qualitative study

#### Domains identified through literature review

The literature review indicated that non-motor symptoms have a strong impact on the HRQOL of PD patients [39-42], and that they are associated with experiencing off-time [19,21]. Based on these findings, the following symptoms were anticipated domains for discussion in the focus groups of PD patients with off-time: feelings of anxiety, mood swings, loss of interest, fatigue and autonomic or gastrointestinal symptoms (such as excessive sweating, salivation, and incontinence).

#### Characteristics of focus groups participants

Patients who participated in the focus groups were mostly white (82%), male (67%) and retired (70%). Most experienced off-time between 1% and 25% of the day (75%). Education level varied among the participants, from high school or GED (6%) to graduate degree (18%). Nearly half (45%) had been diagnosed with PD for more than 5 years.

#### Content validity of original SCOPA-DC in the U.S. population

All but two of the items in the original SCOPA-DC were intuitive and well comprehended by PD patients. The uncontrollable movements item caused a limited amount of confusion for some patients, indicating that the wording of this item may need to be modified. However, difficulties with this item were not constant across focus groups. Further, many patients had difficulty understanding the way the multi-part sleep item was presented. Finally, many patients felt that the instructions could be clarified, the day segment labels removed, and the response options streamlined.

#### Domain elicitation

Patients spoke about the emotional effects of PD and identified non-motor symptoms that interfered with their ability to complete daily activities or to engage in work or social situations. Patients commented on their inability to complete almost any activities due to the unexpected and overwhelming effect of fatigue and described how the physical challenges of being in public were often the precursor to feelings of anxiety. Feelings of frustration over the inability to recall simple facts and to retain recent information indicated problems in the areas of concentration and memory. Patients also described how they would be awakened by sudden pains during the night or when resting. Autonomic symptoms such as difficulty swallowing, having to take frequent and uncontrollable restroom breaks, and excessive sweating as a result of very simple tasks such as walking while shopping were also frequently mentioned by patients.

There was strong endorsement of these symptoms appearing in association with off-time episodes. Patients explained how off-time experiences varied widely in terms of place (at home, while driving, while shopping), time of the day, and symptoms. While motor symptoms were the most noticeable, non-motor symptoms were also strongly identified as occurring specifically with off-time, and going away after the off-time episode had passed. Patients believed they could reliably tell when they were experiencing off-time, and that their physicians and nurses had taught them about off-time early on in their treatment.

#### Changes to original SCOPA-DC

Based on the findings of the 3 focus groups, a Revised SCOPA-DC instrument was created by: 1) modifying the instructions and labels for day segments and response options as well as the format of the sleep item; 2) the addition of 7 non-motor symptom items (fatigue; memory; anxiety; pain; difficulty swallowing; urgent urination; sweating); and 3) replacement of the X's (within boxes) with circles around the numbers to denote the patient's responses. A single item assessing off-time (yes/no) at each time point was included for validation purposes.

Participants of the cognitive debrief indicated that the Revised SCOPA-DC was an improvement over the original SCOPA-DC. First, they felt that the new format was easier to use as they were better able to focus on the items and select a valid response for each time frame. Furthermore, participants felt that the original SCOPA-DC did not adequately capture their experiences with PD throughout the day and they valued the addition of the non-motor symptoms. During the cognitive debrief patients indicated that all non-motor symptoms added to the Revised SCOPA-DC were relevant and related to off-time experiences, suggesting good content validity.

### Phase II - psychometric evaluation of the Revised SCOPA-DC

#### Recruitment

Based on screening questions, 401 PD patients were identified as being eligible to participate in the study. Among these 401, 165 (41%) consented to be in the study, answered all required questions and were mailed the Revised SCOPA-DC; 101 (61%) returned completed forms for the Revised SCOPA-DC.

#### Sample characteristics

The mean (SD) age of patients was 66.3 (12.5) years (Table 1). Half (50.5%) were male, and the vast majority were white (88.1%). Most (80.2%) had been diagnosed with PD for one year or longer (average = 7.4 years). Sixty-one percent of patients were taking levodopa at the time they answered the survey and 82.3% of these had been taking it for at least one year.

**Table 1:** Sample Characteristics (N = 101)

		N	(%)
Age in years, mean (SD)		66.3	(12.5)

Gender	Male	51	(50.5)

Education	Less than high school	6	(5.9)

	High school	13	(12.9)

	Some college	41	(40.6)

	Bachelor's degree or higher	41	(40.6)

Race/Ethnicity	White, Non-Hispanic	89	(88.1)

	Black, Non-Hispanic	5	(5.0)

	Hispanic	4	(4.0)

	Other	3	(2.9)

Marital Status	Married	71	(70.3)

	Widowed	6	(5.9)

	Divorced/Separated	13	(12.9)

	Never Married/Living with partner	11	(10.9)

Employment Status	Working - paid employee	20	(19.8)

	Working - self-employed	1	(1.0)

	Not working/Retired	80	(79.2)

Diagnosed with PD	Less than 1 year	20	(19.8)

	1 year or more	81	(80.2)

	Number of years, mean (SD)	7.4	(5.4)

PD Symptoms	Resting tremors	78	(77.2)

	Slowed ability to start and continue movements	41	(41.0)

	Rigidity or inability to complete a movement, stiffness	35	(35.0)

	Difficulty with balance or instability	51	(51.0)

	Stooped, forward-leaning posture	32	(32.0)

	Freezing or sudden, brief inability to move the feet	16	(16.2)

Daily Off-Time	None	15	(14.9)

	1-25% of the day	58	(57.4)

	26-100% of the day	28	(27.7)

Currently taking levodopa		62	(61.4)

Time taking levodopa	Less than 1 year	11	(17.7)

	1 year or more	51	(82.3)

PDQ-8 Summary Index, mean (SD)		34.3	(22.5)

WOQ-9 Percentage of Symptoms, mean (SD)		60.5	(22.2)

SF12v2 - Physical Component Summary, mean (SD)		38.2	(10.7)

SF12v2 - Mental Component Summary, mean (SD)		46.0	(10.5)

PDQ8-SI = Parkinson's Disease Questionnaire-8 Summary Index; WOQ-9 = Wearing-Off Questionnaire-9; SF-12 = Short Form-12 Health Survey; PCS = Physical Component Summary;

MCS = Mental Component Summary; SD = Standard deviation; PD = Parkinson's disease.

Patients who completed the study (N = 101) differed from those who did not return the complete diary (N = 64) only with respect to employment status; retirees made up a larger proportion of the former group (63% versus 48%). No other statistically significant differences were found between completers and non-completers with respect to the characteristics shown in Table 1.

#### Factorial structure of the Revised SCOPA-DC

CFA models for the hypothesized 2-factor structure resulted in goodness of fit indices that remained above the desired cutoff values for acceptable model fit. Model refinement was undertaken by excluding items with lower loadings (items 4 and 9) and by allowing residual error correlations to be estimated between items 6 and 7, but goodness of fit indices remained above the recommended values of moderate fit (Table 2).

**Table 2:** Standardized Factor Loadings from Alternative Confirmatory Factor Analyses Models

Revised SCOPA-DC Item	Two-Factor Model^┼^		Three-Factor Model		
	**Factor 1**	**Factor 2**	**Factor 1** **(Mobility)**	**Factor 2** **(Physical Functioning)**	**Factor 3** **(Psychological Functioning)**

01. Walking	0.82		0.79		

02. Changing position	0.92		0.98		

03. Using your hands	0.68			0.74	

04. Uncontrollable movements	-			0.67	

05. Feelings of exhaustion or fatigue		0.80		0.77	

06. Difficulty concentrating or remembering		0.76			0.95

07. Feelings of anxiety or panic		0.59			0.75

08. Unexplained pains		0.78		0.80	

09. Difficulty swallowing		-		-	

10. Frequent or urgent urination		0.69		0.72	

11. Sweating too much		0.58		0.56	

CFI/TLI	0.94/0.91		0.97/0.96		

RMSEA (90% confidence interval)	0.09 (0.05, 0.13)		0.06 (0.00, 0.10)		

SRMR	0.06		0.05		

^┼ ^This model was obtained after model refinement: items 4 and 9 were excluded; items 6 and 7 were allowed to be correlated.

CFI = Comparative Fit Index, TLI = Tucker-Lewis Index, RMSEA = Root Mean Square Error of Approximation, SRMR = Standardized Root Mean Residual; SCOPA-DC = Scales for Outcomes of Parkinson's disease Diary Card. Threshold values indicating good fit: CFI and TFI: ≥ .95; RMSEA: < .06; SRMR: < .09.

EFA was then undertaken to determine whether a different domain structure would better represent the measurement model of the Revised SCOPA-DC. A 3-factor structure was found to be a better fit, as indicated by sizeable reductions in the values of goodness of fit indices, and a CFA was conducted with the domain specification shown in Table 2. The final domain structure excluded item 9 (difficulty swallowing), which failed to achieve a loading ≥ 0.40 in the majority of cross-validation runs. All goodness of fit indicators suggested that the 3-factor model was a good fit to the data (CFI/TLI = 0.97/0.96; RMSEA = 0.06; SRMR = 0.05). No modification indices above 10 were observed.

In the final 3-factor structure, factor 1 consists of the walking and changing position items, both of which may be seen as related to mobility impairments. The items in factor 2 may be seen as representing symptoms that interfere with common daily activities and assess general physical functioning, but do not necessarily involve mobility. The items in factor 3 - difficulty concentrating or remembering and feelings of anxiety or panic - are distinct from the remaining items in that they fall strictly into the sphere of psychological (rather than physical) impairment.

#### Item-level psychometrics

Item-scale correlations, which ranged between 0.59 and 0.83, indicated that each item was more strongly correlated with the total score of the hypothesized domain than with the total scores of either of the two remaining domains (Table 3), supporting the proposed model. Cronbach's Alpha values obtained after removing an item from the Physical Functioning domain indicated that most items contributed similarly to this scale.

**Table 3:** Item-Scale Correlations Corrected for Overlap and Reliability Statistics for Three-Factor Model

	Mobility	Physical Functioning	Psychological Functioning	Cronbach's Alpha	Cronbach's Alpha-item deleted
MOBILITY				0.87	

01. Walking	**0.77**	0.59	0.51		^┼^

02. Changing position	**0.77**	0.67	0.55		^┼^

PHYSICAL FUNCTIONING				0.86	

03. Using your hands	0.63	**0.81**	0.50		0.83

04. Uncontrollable movements	0.37	**0.72**	0.53		0.84

05. Feelings of exhaustion or fatigue	0.61	**0.83**	0.65		0.83

08. Unexplained pains	0.63	**0.74**	0.56		0.82

10. Frequent or urgent urination	0.43	**0.77**	0.56		0.83

11. Sweating too much	0.35	**0.59**	0.45		0.86

PSYCHOLOGICAL FUNCTIONING				0.83	

06. Difficulty concentrating or remembering	0.55	0.69	**0.72**		^┼^

07. Feelings of anxiety or panic	0.47	0.56	**0.72**		^┼^

Model Based Reliability	0.88	0.87	0.86		

^┼^Cronbach's Alpha if item is deleted is not meaningful for a 2-item subscale.

#### Reliability

All three subscales showed good to excellent Cronbach's Alpha values (Table 3), confirming the reliability evaluated based on CFA model parameters.

#### Convergent and discriminant validity

The subscales of the Revised SCOPA-DC were generally more strongly correlated with the PDQ-8 and the SF-12v2 than with the WOQ-9 (Table 4). The correlation between the Mobility subscale scores and PCS (0.54) scores were greater than those with MCS scores (0.47). Conversely, the correlation between the Psychological Functioning subscale scores and MCS (0.58) scores were greater than those with PCS scores (0.39). However, contrary to what was hypothesized, the Physical Functioning scores were more strongly correlated with MCS (0.60) scores than with PCS (0.46). These results are suggestive of good convergent validity for all 3 Revised SCOPA-DC subscales and good discriminant validity for 2 Revised SCOPA-DC subscales.

**Table 4:** Convergent/Discriminant and Known-Groups Validity

	Convergent/Discriminant Validity (Spearman Correlation Coefficients)				Known-Groups Validity Mean (SD)			
	
	HRQOL Measures				Baseline Off-Time		Disease Duration	
Revised SCOPA-DC Subscale	PDQ8-SI	WOQ-9 Percentage of Symptoms	SF-12 PCS	SF-12 MCS	Absence of off-time	Presence of off-time	Up to 5 years	More than 5 years

Mobility	0.60^†^	0.45^†^	-0.54^†^	-0.47^†^	21.7 (17.3)^1^	28.3 (21.7)	24.9 (21.1)^3^	30.4 (21.1)

Physical Functioning	0.58^†^	0.56^†^	-0.46^†^	-0.60^†^	16.0 (17.6)^1^	21.0 (15.5)	19.3 (15.6)^3^	20.5 (14.7)

Psychological Functioning	0.62^†^	0.48^†^	-0.39^†^	-0.58^†^	9.2 (20.7)^2^	15.9 (17.8)	12.4 (17.4)^3^	14.9 (15.8)

PDQ8-SI = Parkinson's Disease Questionnaire-8 Summary Index; WOQ-9 = Wearing-Off Questionnaire-9; SF-12 = Short Form-12 Health Survey; PCS = Physical Component Summary; MCS = Mental Component Summary; SD = Standard deviation; SCOPA-DC = Scales for Outcomes of Parkinson's disease Diary Card; HRQOL = Health-Related Quality of Life.

† = p < 0.001.

^1. ^Not statistically significantly different from mean score of fluctuators (p > 0.05).

^2. ^Statistically significantly different from mean score of fluctuators (p = 0.023).

^3. ^Not statistically significantly different from mean score of > 5 years (p > 0.05).

#### Known-groups validity

Mean scores on all three subscales of the Revised SCOPA-DC (Table 4) were lower for patients who reported experiencing no off-time than for patients who reported experiencing off-time on a normal day, at baseline. Similarly, all three means were lower among patients who were diagnosed with PD up to 5 years prior to the study compared to those who had been diagnosed with PD for more than 5 years. However, none of these differences reached statistical significance, except for the Psychological Functioning subscale when using off-time as the criterion variable (the average score for patients with off-time was 6.7 points higher than for patients without off-time; p = 0.023).

#### Measurement of off-time

There were no significant differences between patients with and without baseline off-time in mean CV scores based on the Mobility or Physical Functioning subscales (Table 5). Significance testing was not evaluated for the Psychological Functioning due to insufficient sample size (N = 2 in the absence of off-time group). Using an alternative measure of symptom variability, patients with baseline off-time had significantly higher SDs on the Psychological Functioning (0.65 versus 0.33; p = 0.018) and Physical Functioning (1.73 versus 1.32; p = 0.053) subscales as compared to those without baseline off-time.

**Table 5:** Comparison of Symptom Fluctuations on the Revised SCOPA-DC Subscales Across Patients with and without Baseline Off-time

	Absence of off-time		Presence of off-time		
	
	N	Mean	N	Mean	P-value
Mobility					

CV	8	0.56	53	0.50	0.477

SD	15	0.76	86	0.90	0.275

Physical Functioning					

CV	9	0.53	70	0.51	0.908

SD	15	1.32	86	1.73	0.053

Psychological Functioning					

CV	2	0.60	31	0.60	*

SD	15	0.33	86	0.65	0.018

CV = Coefficient of variation; SD = Standard deviation.

* Not tested given insufficient sample size (N < 5).

#### Prediction of off-time

All 3 Revised SCOPA-DC subscales performed in a similar manner with respect to their ability to predict the presence of off-time (Table 6), as captured at each time period. The odds of experiencing off-time were approximately 30% (Physical Functioning subscale) to 50% (Mobility subscale) higher for patients with a 1-point higher score. About two thirds of the observations were correctly classified by each of the subscales.

**Table 6:** Estimated Coefficients for GEE Logistic Regression Predicting the Probability of Off-time

Revised SCOPA-DC Subscale	Model Parameter (SE)	Odds Ratio (95% CI)	Chi-Square	P-value	Percentage of Correctly Predicted Observations
Mobility	0.40 (0.07)	1.49 (1.30-1.71)	26.80	< 0.0001	69.0%

Physical Functioning	0.25 (0.04)	1.28 (1.19-1.39)	32.20	< 0.0001	67.7%

Psychological Functioning	0.30 (0.07)	1.35 (1.18-1.55)	16.76	< 0.0001	69.1%

GEE = Generalized Estimating Equations; SE = Standard Error; CI = Confidence Interval; SCOPA-DC = Scales for Outcomes of Parkinson's disease Diary Card.

## Discussion

This study aimed to evaluate the validity of a modified version of the SCOPA-DC [22], a diary card originally developed in the Netherlands to measure motor disability among PD patients with symptom fluctuations. Literature review and qualitative findings indicated support for the addition of non-motor symptoms and changes to the format and wording of response choices of the original SCOPA-DC. Based on these findings, a Revised SCOPA-DC was developed and subsequently administered to a sample of non-institutionalized U.S. subjects with self-reported PD. Factor analysis indicated that the measurement model of the Revised SCOPA-DC was best represented by a 3-factor structure. The first factor (Mobility) tapped into issues of mobility (walking and changing position), the second factor (Physical Functioning) included 6 items that covered a broader range of symptoms, including impairment in fine motor skills (using your hands, uncontrollable movements), autonomic dysfunction (frequent or urgent urination and sweating too much) and other well recognized PD symptoms such as pain (unexplained pains), and fatigue (feelings of exhaustion or fatigue), while the third factor (Psychological Functioning) addressed psychological factors (difficulty concentrating or remembering and feelings of anxiety or panic). One item (difficulty swallowing) was excluded due to consistently weak factor loadings.

Correlations between the Revised SCOPA-DC and other HRQOL scores indicated good convergent validity. Contrary to what we had hypothesized, correlations between the Revised SCOPA-DC and PD-specific measures were not higher than correlations with the SF-12v2. Although statistical significance was not always achieved, findings from known-groups validity analyses indicated that scores on the Revised SCOPA-DC were lower among participants whom did not report experiencing baseline off-time when compared to those whom reported experiencing off-time, further supporting the construct validity of the revised instrument.

Due to the relative scarcity of high severity ratings on certain Revised SCOPA-DC items, CV scores could not be evaluated for a number of patients, yielding a set of values with insufficient variation to effectively test the validity of this measure. Nevertheless, patients whom reported experiencing off-time at baseline, did have, on average, higher SD values in all three subscales, than patients without off-time, with two of these differences being statistically significant. All three subscales performed satisfactorily with respect to their ability to predict off-time (Mobility and Psychological Functioning: 69%; Physical Functioning: 68%).

The end-of-study feedback questionnaire indicated that study participants had a very positive experience using the Revised SCOPA-DC. Despite a few reports of uncertainty over whether the 3-day period was sufficient to capture periods of off-time, patients were extremely receptive at the idea of using the Revised SCOPA-DC during and beyond the study period. The written comments and numerical ratings indicated that the content of the Revised SCOPA-DC was meaningful to these patients, that the form was easy to complete and did not impose an excessive burden on their daily routine.

Some study limitations should be noted. First, online recruitment of patients could have introduced a bias because individuals lacking appropriate skills and/or resources were not invited to participate. Second, the diagnosis of PD was self-reported which could have caused misclassification. Third, a standardized scale of non-motor symptoms was not administered, which prevented further testing of convergent validity. Finally, although we used various methods to assess the robustness of results, it is possible that goodness of fit statistics were below the desired thresholds as a result of the size of the sample, an effect that has been previously reported [43]. It is important to keep in mind that this could have led to the better performance of the 3-factor structure over the pre-hypothesized motor versus non-motor 2-factor structure. Although our results indicated the 3-factor model to be a better fit to our data, the PD literature most often classifies symptoms as motor or non-motor, which is in alignment with the 2-factor structure. Thus, additional research is needed to test whether the results presented in the current study can be generalized to other samples of PD patients.

Despite increasing awareness that non-motor symptoms may have a greater impact on the HRQOL of PD patients than motor symptoms [7,44], the number of studies that have concurrently evaluated the full spectrum of non-motor symptoms is small. Until recently, the evaluation of the wide range of non-motor symptoms in PD required a large number of tools. This may explain the relative paucity of comprehensive assessments of both motor and non-motor symptoms in PD, both in observational studies as well as studies involving treatment efficacy assessments. As a result, recent efforts [4,17,45,46] have been made to create questionnaires that provide a unified assessment of non-motor and motor PD symptoms severity, but none of these instruments were designed for multiple daily self-reported assessment. For example, although the Unified Parkinson's Disease Rating Scale (UPDRS) was revised and expanded [46] to reflect a greater focus on non-motor symptoms, it is not meant to be entirely answered by the patient and still requires physician input. Thus, to our knowledge, the Revised SCOPA-DC (Additional File 1: Appendix) fills an important gap in the assessment of PD symptoms.

## Conclusions

The results of the current study provided preliminary evidence of the domain structure of the Revised SCOPA-DC. Although use of the Revised SCOPA-DC in future studies is needed to confirm the results encountered in our study, our findings indicated that the Revised SCOPA-DC is a valid and reliable instrument for measuring the impact of PD symptoms and the severity of off-time. Longitudinal studies that allow for the assessment of specific properties such as test-retest reliability and responsiveness will provide further insight into other aspects of the Revised SCOPA-DC that could not be evaluated in the current study. Furthermore, future studies should continue to examine the instrument's domain structure, its ability to measure the severity of symptom fluctuations and to explore alternative measures of variation that can be applied to the entire range of PD severity.

## List of abbreviations

CFA: Confirmatory factor analysis; CFI: comparative fit index; CV: coefficient of variation; EFA: exploratory factor analysis; GEE: generalized estimating equations; HRQOL: Health-Related Quality of Life; KN: Knowledge Networks; MCS: Mental Health Component Summary; NEIRB: New England Institutional Review Board; PCS: Physical Component Summary; PD: Parkinson's disease; PDQ-8: Parkinson's Disease Questionnaire-8; RMSEA: root mean square error of approximation; SCOPA-DC: Scales for Outcomes of Parkinson's disease Diary Card; SD: standard deviation; SE: Standard Error; SF-12v2: SF12v2 Health Survey; SRMS: standardized root mean residual; TLI: Tucker-Lewis Index; UPDRS: Unified Parkinson's Disease Rating Scale; WLSMV: weighted least squares means and variance adjusted; WOQ-9: Wearing Off Questionnaire-9.

## Competing interests

POB and JCH are employees of Teva Neuroscience, Inc., the sponsor of this study. RRB and MKW have served as consultants for Teva Neuroscience, Inc.

## Authors' contributions

RRB co-led the organization and execution of the validation project, conducted the statistical analyses, and contributed to writing and revising the manuscript. POB conceptualized the validation project, assisted with the organization and execution, and contributed to writing and revising the manuscript. MKW co-led the organization and execution of the validation project and contributed to writing and revising the manuscript. JCH conceptualized the validation project and contributed to writing and revising the manuscript. All authors read and approved the final manuscript.

## Supplementary Material

Additional file 1 **Appendix: Revised SCOPA-Diary Card**. Click here for file
