# Supplementary material for: Psychometric validation of the revised SCOPA-Diary Card: expanding the measurement of non-motor symptoms in parkinson's disease
Source: Health Qual Life Outcomes. 2011 Aug 18;9:69. doi: 10.1186/1477-7525-9-69 (PMC3173285; doi:10.1186/1477-7525-9-69)
Supplement: Additional file 1 — Appendix: Revised SCOPA-Diary Card. [file 1477-7525-9-69-S1.DOC]

Appendix. Revised SCOPA-Diary Card

Revised with permission from Marinus J, Visser M, Stiggelbout AM, Rabey JM, Bonuccelli U, Kraus PH, van Hilten JJ: Activity-based diary for Parkinson's disease. *Clin Neuropharmacol* 2002, 25(1):43-50. For inquiries about this adaptation, please contact Philip O. Buck at philip.buck[@tevapharm.com](mailto:rfarkouh@rti.org)
